# Unveiling the Anti-Aging Potential of Marine Natural Bioproducts

**DOI:** 10.3390/md23040165

**Published:** 2025-04-11

**Authors:** Nedeljka Rosic

**Affiliations:** 1 Faculty of Health, Southern Cross University, Gold Coast, QLD 4225, Australia; nedeljka.rosic@scu.edu.au; 2 Marine Ecology Research Centre, Southern Cross University, Lismore, NSW 2480, Australia

**Keywords:** algae, bioactive compounds, anti-aging, skin care, proteomics

## Abstract

Aging is a natural process resulting in the progressive impairment of multiple functions in the human body, leading to a decline in cellular functionality and the development of aging-related diseases. External stress factors, such as ultraviolet (UV) radiation, pollution, and toxin exposure, increase oxidative stress, damage cellular repair mechanisms, and speed up aging processes. With the rise in the world’s aging population, there are enlarged demands for the use of sustainable natural products in food, nutrient supplements and cosmetics that can slow down aging and prolong healthy life and longevity. Algae, including both macroalgae and microalgae, have been recognised as a source of valuable proteins, amino acids, fatty acids, vitamins, and minerals useful for human consumption and medical applications. With increasing demands for nutraceutical and pharmaceutical bioproducts from environmentally friendly resources, the biotechnological industry, over recent decades, has had to provide new, advanced solutions using modern high-throughput omics technologies. The application of proteomics in the area of discoveries of natural products with anti-aging properties has become more popular for wide industry applications. New proteomics profiling provides a better understanding of changes occurring in protein and peptide content, their structure, function and interactions, as well as the regulatory processes and molecular pathways. Mass spectrometry-based proteomics has been used for a wide range of applications including protein identification, characterisation, as well as quantification of proteins within the proteome and sub-proteome. The application of chemical proteomics facilitated the identification of natural products approach and included the synthesis of probes and target fishing, allowing the advanced identification of proteins of interest. This review focuses on marine macro- and microalgal anti-aging compounds and novel proteomics approaches, providing recent experimental evidence of their involvement in anti-aging processes that should facilitate their use in innovative approaches and sustainable biotechnological applications.

## 1. Introduction

Natural compounds, including their structural analogues, historically played an important role in drug discovery and in treating various infectious diseases, as well as other malicious conditions [1]. In civilizations worldwide, natural products have been used as a source of food, water, and energy since ancient times [2]. The interest in applying natural bioactive compounds in skin care products has also risen, along with the availability of marine ingredients [3]. Global industry development and an increase in the world population, with more than 1 billion people living in highly nature-dependent tropical regions, has resulted in increasing demands for natural products worldwide [4]. As the world population is estimated to rise to nearly 10 billion people by 2050, higher demands for natural resources, specifically food resources, are anticipated [5].

The demand for anti-aging products has tremendously increased over the past decade, with a global market valued at more than USD 48 billion [6]. The forecast analyses of future trends in the anti-aging product market for 2025–2033 predict further growth, reaching USD 82 billion by 2033 [6]. With increasing awareness of aging processes and the benefit of using anti-aging products, especially for skin care [7], plus the estimated rise in the world’s elderly population, reaching 2.1 billion by 2050 [8], the need for anti-aging products is likely to grow exponentially in the future.

The marine environment covers more than 70% of the planet and is the world’s largest ecosystem, encompassing extreme conditions, including high salinity, very low and high temperatures, and low and high light conditions [9,10]. With limited land and freshwater availability and further climate challenges, new environmentally sustainable resources are required, and the advanced use of algae presents a green industry of the future that could minimise the use of fossil fuels [11,12]. The biodiversity of marine organisms has been used as a rich source of natural products, extracted from microorganisms and phytoplankton, red, green, and brown macroalgae, and animals such as sponges, cnidarians, molluscs, tunicates, echinoderms, and other marine groups [13]. The application of marine algae and its derivatives has been recognised as cheap, freely available, easily scalable, and capable of meeting the rising needs of natural products [1,14]. The sustainable advantages of using algae for mass production include lowering CO_2_ emissions, reducing the use of limited agricultural lands, generating products with high protein content and other nutritious values, and protecting biodiversity [5]. Algae are photosynthetic organisms capable of successfully growing in diverse environmental conditions, demonstrating phenotypic plasticity and the ability to endure and adapt to stressful environmental conditions such as extreme temperatures and UV radiation [9,15,16,17,18]. Algae, classified into multicellular macroalgae and unicellular microalgae, are recognised for their various biotechnological applications, such as in human and animal food, as a source of bioactive compounds with rich therapeutic properties important for new drug discoveries, as well in other industry applications and innovations [19,20].

Macroalgae, known as seaweed, are found in marine and freshwater environments and have diverse potential in biotechnology [21]. Based on their pigmentation and chemical signature, marine macroalgae can be divided into three main groups: brown seaweed (Phaeophyceae), red seaweed (Rhodophyceae), and green seaweed (Chlorophyceae). Since ancient times, macroalgae have been used in human and animal foods across the globe, from Asia to America [22]. Recently, due to promising nutritional and pharmaceutical values, with rich content of proteins and other promising bioactive compounds, macroalgae applications extended toward various pharmacologically critical applications in multiple industries, from bioremediation to other ecological roles [23,24]. Therefore, seaweeds have been produced on a larger scale in some Asian countries due to increasing demands for marine natural products [25].

Microalgae, single-cell photosynthetic microorganisms, are genetically diverse and found in marine, freshwater, and soil ecosystems [26]. These unicellular organisms can rapidly and flexibly grow in a wide range of conditions [27]. Consisting of diverse valuable products, microalgae have been used in the human diet [26] as a source of drugs and nutrients [28,29,30]. From a sustainable perspective, microalgal species present a promising alternative to fossil fuels as a new biofuel, while using lower environmental resources and being capable of mass production in even marginal areas using wastewater [31]. Multiple microalgal species, such as *Chlorella* sp. and *Arthrospira* sp., have a high protein content as a sustainable source of edible proteins for food consumption [5,32], while others have been recognised for biofuel potential [33,34]. The recent development of new technologies and approaches has aimed to increase the industry application of micro-algae, for example, to improve protein digestibility [5] and its use in human food [20]. However, consumer acceptance still presents some challenges [35]. The application of polysaccharides from microalgae has been targeted to be improved by enhancing its production, extraction, and purification conditions, specifically targeting exopolysaccharides due to their pharmacological properties [36]. Other biotechnological applications of microalga include the use of phenolic compounds and carotenoids [37], sources of vitamins [29], as well as for reducing carbon footprints [12] and biofuel production [27,33].

**Figure 1:**
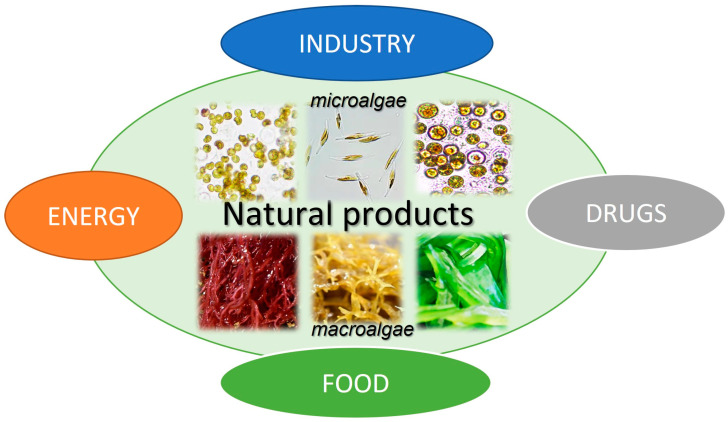
Applications of natural marine products in the 21st century.

The majority of marine ingredients that are used in anti-aging cosmetics products come from algae, including red, brown, and microalgae [3]. Consequently, macro- and microalgal species have shown great potential as sources of natural products for further applications in cosmetics, anti-aging products, and other biotechnological applications (Figure 1). The natural process of aging leads to the body’s functional qualities gradually deteriorating, lowering fitness and increasing the risk of developing chronic illnesses [35]. Degeneration of the main body functions occurs, and aging is sped up by exposure to environmental stressors (e.g., UV radiation, toxin exposure), hereditary factors, and other external factors [36]. Human skin, beyond its role in protection, presents the largest organ that exists, and with aging, it loses its elasticity and moisturising ability [3,37]. Damaging UV radiation, pollution, and exposure to other environmental toxins are examples of external stressors raising oxidative stress, harming cellular repair systems, accelerating cellular death, and promoting aging [15,38,39]. As the world’s population ages, there is a greater need for natural solutions that can be applied topically or consumed through diet to slow down the aging process and extend longevity. These contemporary developments are putting more and more pressure on the biotechnology sector to employ cutting-edge, high-throughput omics technologies and offer novel solutions using eco-friendly, sustainable resources.

In this review, the aims were (1) to discuss recent biotechnological trends in anti-aging natural products from marine algae, (2) to provide an overview of new movements in natural anti-aging products, and (3) to evaluate the use of modern technology, such as proteomics in discoveries of anti-aging natural products.

## 2. Aging

**Figure 2:**
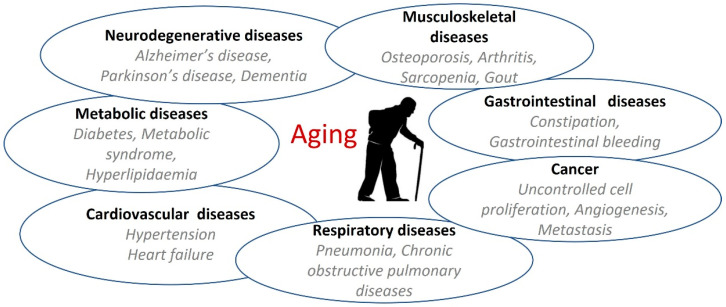
The schematic overview of aging-related diseases.

Aging is a natural and complex process that includes the incremental over-time accumulations of errors in cellular structures, tissues, and organs, leading to a decrease in body fitness and the development of chronic diseases [40]. The number of age-related diseases will continue to rise with the constant increase in the proportion of the aging population (Figure 2). The major factors contributing to the development of aging include internal factors such as genetic predisposition and telomere shortening and also exposure to certain detrimental external factors like toxins and radiation, which have been found to cumulatively impact the progression of aging [41]. Specific signs of aging include molecular signs (e.g., genomic instability, epigenetic changes, mitochondrial dysfunction), as well as cellular (e.g., cellular senescence) and systemic changes [35]. Genetic factors have been proposed to contribute to around 25% of the variability in human longevity [42], with more than 50 genes (e.g., apolipoprotein E gene and gene *FOXO3* [43]) identified via genome-wide studies to have implications for human aging and longevity, with epigenetic changes also playing a key role in regulatory cellular processes of aging [44]. Accumulation of senescent cells and telomere shortening are also recognised intrinsic factors of aging [45]. Telomeres, as repetitive DNA sequences, are found at the end of chromosomes, and they have a protective role in preserving coding parts of the chromosomes during cell replication [46]. As cells proliferate, telomere shortening occurs over time, limiting the number of possible cell divisions and later resulting in mitochondrial dysfunction, genome instability, apoptosis, accumulation of senescent cells, and aging [45,47]. Oxidative stress due to the accumulation of reactive oxygen species (ROS) also leads to genome instability due to damaging impacts on DNA and other macromolecules, contributing to telomere dysfunction and cellular senescence [48]. The immune system declines with aging, resulting in chronic inflammation and a lowering of the body’s ability to fight infection [40]. Geriatric syndromes and chronic diseases such as cardiovascular, osteoarthritis, diabetes, and neurodegeneration following dementia, Alzheimer’s, and Parkinson’s diseases are commonly developed in aging populations [49].

## 3. Anti-Aging Compounds

Due to beneficial pharmacological activities, various anti-aging compounds from natural resources have been explored [3,36,50]. A number of algal species have shown a potential to be used in cosmetics, presenting cost-effective and sustainable opportunities to be utilised in biotechnology [3]. Metabolites obtained from marine resources such as seaweeds and microalgae demonstrated significant potential anti-aging properties for use in cosmetics and other applications (Table 1). Bioactive compounds and their derivatives were shown to delay aging and prolong life via maintaining redox homeostasis, reducing oxidative stress, anti-inflammatory actions, and UV-absorbing properties [50,51]. Useful anti-aging compounds found in algae comprise polyunsaturated fatty acids, vitamins, polyphenols, mycosporine-like amino acids, and trace elements and minerals (Table 1). These diverse bioactive molecules are often found as a mixture of beneficial anti-aging compounds in various algal species, such as brown algae kelp, that enhance wound healing and skin regeneration [52] or red macroalgae, like *Porphyra linearis* and *Phycocalidia acanthophora*, that contain antioxidants and anti-inflammatory and UV-absorbing molecules [53]. Another red macroalgae, *Hypnea musciformis*, which is widely distributed around the Mediterranean Sea, contains a range of pharmacologically promising bioactive molecules (i.e., polysaccharides, flavonoids, and phlorotannins) with anticancer effects reported for the liver and intestinal cancer cells [54,55]. Red seaweed *Asparagopsis taxiformis* extracts have antioxidative, antiviral, and anticancer properties [56], as well as anti-methanogenic activity that is found to be useful for the reduction and mitigation of methane-associated climate change [57].

### 3.1. Polyunsaturated Fatty Acids

Polyunsaturated fatty acids (PUFAs) encompass omega-3 (n-3) and omega-6 (n-6) fatty acid classes and are essential nutrients that are used in human and animal food [58]. Very long PUFAs (20 or 22 carbon chains) from algae are of particular industry interest [59]. PUFAs were reported in microalgal species such as *Chlorella* sp., *Nannochloropsis oculata*, *Pseudochoricystis ellipsoidea*, and *Botryococcus braunii* [60,61], as well as various seaweed species coming from green, red, and brown macroalgae [62,63]. Eicosapentaenoic acid (EPA) and docosahexaenoic acid (DHA) omega-3 fatty acids are beneficial PUFAs that are now successfully sourced from seaweeds instead of fish, which helps in an attempt to protect marine ecosystems from overfishing [64]. In PUFA-rich microalgae *Phaeodactylum tricornutum*, n-3 fatty acids were reported at a concentration of 58 mg/g, which is more than double compared to that in fish (21 mg/g), with beneficial EPA at 53 mg/g, while fish contains 7 mg/g [65]. A combination of n-3 and n-6 PUFAs and their appropriate ratios were determined to be beneficial for reducing body inflammation, as well as cardiovascular and nervous system disorders [62]. PUFAs isolated from various macroalgae (*Ulva lactuca*, *Chondrus crispus*, *Laminaria hyperborea*, *Fucus serratus*, *Undaria pinnatifida*, *Palmaria palmata*, *Ascophyllum nodosum*, *Caulerpa taxifolia*, and *Sargassum natans*) were within the concentration of 2–14 mg/g dry matter (DM) and the n-6: n-3 ratio of around 1, which is within the recommended range of 5 or below [66] for beneficial anti-inflammatory actions and a positive pharmacological impact on nervous and cardiovascular systems [62].

Mechanisms of PUFA actions that contribute to anti-aging processes include maintaining redox homeostasis in vivo, decreasing oxidative stress, reducing telomere shortening and downregulating the antioncogene expression [67]. Multiple studies have shown the benefits of taking PUFAs for prevention and/or treatment such as in the case of age-related musculoskeletal diseases (i.e., sarcopenia, osteoarthritis, and osteoporosis) [68] and neurodegenerative diseases, like Alzheimer’s disease (AD) and Parkinson’s disease (PD) [58]. In the case of sarcopenia, there is an age-related loss of strength and muscle mass caused by oxidative stress and the accumulation of ROS in the mitochondria of skeletal muscle cells, leading to mitochondrial dysfunction, and speeding up telomere depletion and cellular senescence [69]. In human studies conducted on older adults, improved skeletal muscle growth and functionality were reported, leading to a lower risk of developing sarcopenia due to the consumption of omega-3 fatty acids compared to the control group [70,71]. In studies in vitro, PUFAs were found to have a cytoprotective effect via lowering the level of ROS by increasing the cellular activities of antioxidative enzymes superoxide dismutase and glutathione peroxidase in cells, resulting in their cytoprotective effect and repair of mitochondrial function [72]. Mechanisms of PUFA action in sarcopenia prevention beyond the reduction of oxidative stress also include reduction of inflammation and improvement of mitochondrial health [68]. Animal studies, in the case of age-related neurogenerative diseases, provided evidence linking higher brain DHA concentration with improved cognitive health, highlighting the importance of PUFAs in neuroprotection and possible prevention of AD and PD [73].

### 3.2. Vitamins

Marine algae accumulate various vitamins, and their precursors with particularly high concentrations of vitamin A (especially precursor β-carotene), but also B1, B2, B6, B12, C, D, and E reported in microalgae in the mature developmental state [28,74], as well as in macroalgal species [21]. Due to rapid growth, microalgae were found to be a potentially very good sustainable source of vitamin D, with additional biotechnological adjustments further enhancing vitamin D production [75]. Exposure to UVB radiation was found to stimulate the synthesis of vitamin D [76], with genetic manipulation and calcium homeostasis also positively impacting vitamin D production under heat stress and nitrogen deprivation [75]. High vitamin quantities were reported per dry weight for *Tetraselmis suecica* (4280 μg/g provitamin A and 6323 μg/g vitamin E), and *Pavlova lutheri* (1162 μg/g vitamin B_12_ and 837 μg/g vitamin C) [77]. Microalgae were also found to be valuable sources of Vitamin E, accumulating tocopherols and tocotrienols forms, useful in cellular protection and chemoprevention [78]. Carnitine is a non-essential amino acid that can be synthesised in the human body from other essential amino acids (e.g., lysine and methionine), and it also needs sufficient levels of vitamins and trace elements as co-factors (e.g., ascorbic acid—vitamin C, ferrous iron, and niacin—vitamin B) [79]. Deficiency in any of these vitamins and trace elements may lead to carnitine deficiencies impacting fatty acid metabolism [80], leading to oxidative stress and aging [81]. There are multiple modes of vitamin actions that contribute to anti-aging processes, including antioxidative action and positive effects on the gut microbiota and gut health [40]. The neuroprotective effect was reported for vitamin E via the reduction of oxidative stress that led to a lower number of p53-positive brain cells [82], while vitamin C combined with some active mixes (containing other bio-peptides, and hyaluronic acid) reduced oxidative stress and stimulated collagen synthesis [83].

### 3.3. Trace Elements and Minerals

During aging, even in healthy elderly people, changes are occurring in the levels of trace elements (TEs), with a drop in levels of zinc (Zn), selenium (Se), and manganese (Mn) and a rise in iron (Fe), copper (Cu), and iodine (I) concentrations [84]. The lower levels of trace elements and minerals were found to be linked with premature signs of aging, such as premature greying, oxidative stress, inflammation, and development of chronic diseases [36,85]. Significantly lower levels of Zn and vitamin D were reported in the blood serum of participants with diagnosed acne conditions compared to the control group [86]. Skin conditions like inflammation and acne vulgaris were found to benefit from Zn supplements [48], indicating the importance of this TE in skin health. Valuable resources of TEs and minerals were found in edible seaweeds with high amounts of macro minerals such as sodium (Na), potassium (K), calcium (Ca), and magnesium (Mg), and TEs (e.g., Fe, Zn, Mn, Cu) [23,87,88]. In the biomass of five different microalgae (*Porphyridium cruentum*, *Isochrysis galbana*, *Phaeodactylum tricornutum*, *Tetraselmis suecica,* and *Nannochloropsis gaditana*), major minerals and TEs were found in good quantities, including other valuable ingredients [89].

### 3.4. Polyphenols

Polyphenols (PPs) are micronutrients found in macro- and microalgae that are used in the human diet [90]. These secondary metabolites are highly hydrophilic and are divided into different groups depending on the phenol ring numbers. PPs such as phenolic acids, phlorotannins, and flavonoids possess beneficial pharmacological properties ranging from antioxidant to anti-inflammatory and anticancer that are important in reducing aging and the impact of age-related diseases [91]. During the aging process, oxidative stress and inflammation may lead to DNA epigenetic modifications, impacting the level of DNA methylation and histone content variations and resulting in changes in gene expression [91,92]. Dietary PPs were found to impact these age-related changes via their antioxidative, anti-inflammatory, and anticancer activities [92]. PPs, such as phlorotannins, isolated from marine brown macroalgae *Laminaria hyperborea*, were found to promote wound healing [93] and to act as antioxidants by scavenging harmful ROS produced under UV radiation [94]. Phlorotannins are a heterogenous group of polymerised phenolic compounds (i.e., phloroglucinol, eckol, fucodiphloroethol G, phlorofucofuroeckol A, 7-phloroeckol, dieckol, and 6,6′-bieckol) [95], which are uniquely found in brown algae and are characterised by high antioxidative potential, as well as anti-allergic, anti-inflammatory, anticancer activity and the role in neuroprotection [96]. The antioxidative activity of phlorotannins was found to minimise UV-induced oxidative stress in large kelps [94], while in extracts isolated from Irish macroalgae *Fucus serratus*, antioxidant capacity was enhanced via the application of reverse-phase flash chromatography fractionation [97]. Flavonoids (e.g., flavones, isoflavones, anthocyanidins, and others) also demonstrate beneficial pharmacological properties, including antioxidant, anti-inflammatory, and anticancer [91], with both microalgae and macroalgae being a rich source of these PPs [98]. PPs are recognised for their anti-viral properties, especially effective on enveloped viruses (i.e., targeting spike and membrane viral proteins), with a potential for being in the pharmaceutical industry for the treatment of viral diseases [99]. In addition, anti-bacterial activities were reported for PP extracts isolated from seaweeds *Corallina officinalis*, *Ulva lactuca*, and *Pterocladia capillacea* [100]. Polyphenol extracts isolated from seaweeds were found to have a beneficial neuroprotective role as antioxidants contributing to the prevention of age-related diseases such as cancer, cardiovascular diseases, arthritis, and other challenges of aging [91], as well as in neurodegenerative diseases such as Alzheimer’s disease and Parkinson’s disease [101]. Ethyl acetate fractions isolated from the brown algae *Padina tetrastromatica* that were tested on human skin cell line (i.e., K-MEL-28) were not toxic at the concentration of up to 100 μg/mL, while demonstrating positive photoprotective and anti-aging potential with a strong sun protection factor (SPF) [102]. Major PPs identified in this ethyl acetate fraction were phlorotannins capable of absorption of UVA and UVB and antioxidative action by mopping up ROS produced under UV exposure [103,104]. PPs isolated from the red macroalgae *Symphyocladia latiuscula* demonstrated a neuroprotective effect when tested on animals with diabetic peripheral neuropathy [105], while edible brown and green macroalgae polyphenol extracts showed high antioxidant activity and moderate anti-bacterial activities [106]. Microalgae are also a promising source of PPs with concentration variation impacted by external conditions [107], indicating the possible biotechnological potential for increasing mass production under controlled cultivation conditions for future sustainable production of PPs.

**Table 1:** Common anti-aging compounds extracted from algae.

Compound Types	Compounds	Example (Molecular Formula, Structure)	Modes of Anti-Aging Action [REF]	Sources (REF)
Polyunsaturated fatty acids (PUFAs)	Omega-3 PUFA Omega-6 PUFA	Eicosapentaenoic acid (C_2O_H_30_O_2_, 20:5) 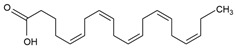	Maintaining the in vivo redox homeostasis; by lowering oxidative stress and reducing telomere shortening; by down-regulating the antioncogene expression [67].	Macroalgae [62] Microalgae [59,108]
Vitamins	Vitamins A, B, C, D, E	Vitamin A (C_20_H_30_O)* 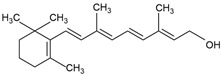 *	Antioxidative; modulating the gut microbiota; improving inetene function; neuroprotective effect; enhancing fat metabolism [50]	Macroalgae [21] Microalgae [29]
Trace elements (TEs) and minerals	Zinc, copper, selenium, sodium, potassium, calcium	Zn, Cu, Se, Na, K, Ca	Maintaining the in vivo cellular homeostasis; metalloenzymes [36]	Macroalgae [87,88] Microalgae [74]
Polyphenols (PPs)	Flavonoids, phlorotannins, phenolic acids, stilbenes, lignans	Phlorotannin: phloroglucinol (C_6_H_6_O_3_) * 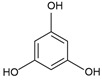 *	Antioxidant; anti-inflammatory; anticancer properties [91]	Macroalgae [99,100,105,106] Microalgae [107,109]
Mycosporine-like amino acids (MAAs)	Mycosporine glycine, shinorine, porphyra-334, mycosporine- 2-glycine, palythine	MAA direct precursor: 4-deoxygadusol (C_8_H_12_O_5_) * 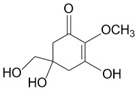 *	UV-absorbing property (max range 310–360 nm); antioxidative; anti-inflammatory; anti-adipogenic [51,110]	Macroalgae [111,112,113,114] Microalgae [115,116,117,118]
Marine algal polysaccharides (MAPs)	Alginate, carrageenan, fucoidan, ulvan, and laminarin	Fucoidan (C_6_H_9_O_3_SO_3_)_n_ * 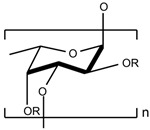 *	Antioxidants; anti-inflammation; antitumor [119,120]	Macroalgae [53,121] Microalgae [122,123]

### 3.5. Amino Acids

Amino acids, the building blocks of proteins, are crucial for cellular signalling and could influence the aging process [124]. Mycosporine-like amino acids (MAAs) are small, low-molecular-weight (<400 Da), water-soluble UV-absorbing molecules that are produced via two pathways, shikimate and/or phosphate pathways [125,126,127,128]. MAAs are found to be temperature and light-stable compounds that exhibit strong photoprotective properties and the capacity to absorb UVA (320–400 nm) and UVB (280–320 nm) light without the production of harmful free radicals [116,129,130,131]. MAAs have high molar extinction coefficients with maximum UV absorption between 310 nm and 362 nm [114,132,133].

From MAA direct precursor, 4-deoxygadusol (4-DG), MAA biosynthetic processes lead to further synthesis of more than 30 various molecules that are divided into primary and secondary MAAs [134]. Selected primary MAAs, commonly studied for biotechnological applications, are presented in Table 2. With UV-absorbance capacities within UVA and UVB ranges, MAAs can effectively release absorbed radiation as heat, lacking the production of ROS [135]. These free radicals such as superoxide anion (O_2_^−^), hydrogen peroxide (H_2_O_2_), and hydroxyl radicals (OH^−^), are also by-products of normal cellular metabolism and, in smaller quantities, they play a role as signalling molecules and in other ordinary physiological processes such as wound healing and repair of tissue [136]. Beyond the role in cellular communication, when present in higher concentration (e.g., in response to UV radiation or other stress factors), elevated levels of ROS may lead to oxidative stress, DNA damage, and cellular aging [137]. Additionally, MAAs have an antioxidative role by mopping up excessive ROS from the body and, therefore, enhancing tolerance to a number of abiotic stress factors [138]. These secondary metabolites are also temperature- and photostable, acting in the prevention of UV-induced thymine dimer formation from damaging DNA molecules [139,140,141]. Therefore, as MAAs play an important role in cellular protection from abiotic stress factors, these molecules have been recognised for the potential to be used in cosmetics for UV skin protection as organic sunscreens [51].

The concentration and profile of MAAs vary in different algal species but are influenced by variations in external environments, including UV levels, temperature, salinity, and nutrients [130,138,142]. For example, seasonal variations in temperature and UV levels were found to impact MAA content and profile in red algae *Iridaea tuberculosis*, *Nothogenia fastigiate*, and *Corallina officinalis*, with MAA content reaching from 0.4 to 1 mg/g of dried mass weight (DW) depending on the season and the species [142]. Nitrogen (N) status was also found to impact the MAA profile, with decreased shinorine levels and increased polythene content in red algae *Asparagopsis armata* grown in tanks when N fluxed was raised [143]. Enriched nutrient conditions (i.e., in nitrogen and phosphorous ions) and elevated UV levels also led to an increase in MAA levels in red macroalgae *Hydropuntia cornea*, *Gracilariopsis longissima*, and *Halopithys incurva* [144]. Generally, the highest MAA levels (above 2 mg/g of DW) were reported in red macroalgae (above 10 mg/g DW) [145] with representative species from the orders Gracilariales, Bangiales, Gelidiales, and Gigartinales [111]. In microalgae, MAA concentration also showed seasonal variation with a positive impact on UV radiation on the accumulation of MAAs [142]. In phytoplankton and copepod *Cyclops abyssorum tantric* sampled from an alpine lake, there was a substantial increase of more than threefold in MAA content during summer compared to winter [146]. The conditions used during MAA extraction and final extract storage were also important for the yield of MAAs and could significantly influence MAA content estimation [147]. In red macroalgae *Phycocalidia acanthophora* and *P. linearis*, the levels of desirable bioactivities varied in different types of extracts (i.e., aqueous extracts vs. the hydroethanolic solution), indicating the importance of extraction procedures for optimisation and improving targeted activities [53].

The anti-aging effects of MAAs have been mainly assessed in primary MAAs, including shinorine, porphyra-334, palythine, and mycosporine-glycine (Table 2). MAAs were confirmed to have anti-inflammatory, antioxidative, and UV-absorbing activities, with anti-aging and skin-firming properties that are beneficial for cosmetic products [51,110,148,149]. MAAs are considered environmentally friendly sunscreens found in edible seaweeds and many other aquatic species [111,116,131,150,151,152]. For assessing anti-aging activities, cellular lines used included human keratinocyte cell line HaCaT and 2,2-diphenyl-1-picryhydrazyl (DPPH) assay [153]. Procollagen levels in human skin fibroblast were evaluated via an ELISA assay [154], while anti-inflammatory and antioxidant effects of MAAs were confirmed on macrophages [155], with immunomodulatory effects [156]. The application of MAAs in cosmetics as more efficient sunscreens with advanced UV-absorbing and anti-aging properties is an exciting area that still needs to be further explored [157,158].

### 3.6. Polysaccharides

Marine algal polysaccharides (MAPs) are essential molecules building cell walls in marine algae [120]. In macroalgae, these macromolecules are quite abundant, reaching from 15% up to 76% of DW [63], while in microalgae, MAPs were reported to make up to 20% of DW [159]. Pharmaceutical activities reported for MAPs include antioxidative, anti-inflammatory, and anti-tumour, which are beneficial for anti-aging, as well as antiviral and hypolipidemic [120]. Polysaccharides are biopolymers made from monosaccharides (i.e., simple sugar) linked with glycosidic bonds. These molecules, beyond being used as food, have a wide industry application, including roles as emulsifiers, thickeners, and stabilisers [160], with some MAPs produced at massive scale (e.g., agar, alginate, starch, and cellulose) [161]. Alginate, isolated mainly from brown seaweeds, showed similarity to extracellular matrices found in living tissue and, for that reason, has been applied in wound healing dressings with enhanced antioxidative capacity when supplemented with silver [162,163]. Fucoidans, a sulphated polysaccharide, isolated from brown seaweeds like *Turbinaria ornata*, have been recognised for anti-inflammatory, antioxidant, and anticancer activities, with anti-photoaging effects [164]. Anti-aging effects of MAPs were confirmed using animal models with antioxidative activity resulting in upregulation of the gene expression of antioxidant enzymes, resulting in the reduction of oxidative stress, as well as regulation of the expression of other age-linked genes and pathways, reduction of telomere attrition and impact on immune modulation [119].

**Table 2:** Primary mycosporine-like amino acids (MAAs); algal sources with representative species and maximum UV absorbance.

Compound Name (Molecular Formula; Structure)	Source of the Compounds [Reference]	UV-Absorbing Maximum (λ_max_)
Shinorine (C_13_H_20_N_2_O_8_) * 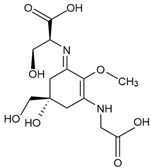 *	Macroalgae Red seaweeds [111,112,114,130,142,145,147,165,166,167] *Rhodymenia* spp., *Acanthophora spicifera* *Gelidium corneum*, *Georgiella confluens; Gelidium amansii*, *Gracilaria confervoides*, *Gracilaria* sp., *Bostrychia scorpioides*, *Porphyra dioica* Brown seaweeds [111,113,168] *Ecklonia radiata*, *Dictyota bartayresii*, *Dictyosiphon foeniculaceus*, *Pilayella littoralis*, *Ecklonia radiata*, *Halopteris scoparia*, *Hydroclathrus clathratus*, *Sargassum oligocystum* Green seaweeds [111] *Prasiola crispa* Microalgae [153,169,170,171] *Chlamydomonas nivalis*, *Cyclops abyssorum tatricus*, *Alexandrium* sp., *Chlamydomonas hedleyi*, *Gloeodinium viscum*, *Gymnodinium catenatum*, *Acetabularia mediterranea* Other MAA sources Cyanobacteria [117,172,173,174] *Nostoc commune*, *Aphanothece halophytica*, *Lyngbya* sp. Fungi and Animals [112,116,175] *Ascochyta pisi*, *Knufia cryptophialidica*, *Emiliania huxleyi*; *Gymnodinium linucheae* Corals, Sea Anemones, Jellyfish	333 nm
Porphyra-334 (C_14_H_22_N_2_O_8_) * 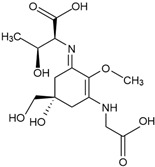 *		334 nm
Mycosporine-glycine (C_10_H_15_NO_6_) 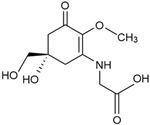		310 nm
Mycosporine-2-glycine (C_12_H_18_N_2_O_7_) 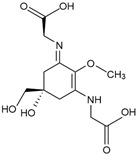		332 nm
Palythine (C_10_H_16_N_2_O_5_) 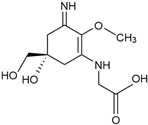		320 nm

## 4. In Vitro, Ex Vivo, and In Vivo Experimental Assessments of Natural Products for Anti-Aging Properties

Experimental in vitro and in vivo studies provided specific evidence regarding the anti-aging activities of NPs of interest [3,6,7,36,50,83,110]. Various in vitro assays have been developed to assess the desired anti-aging activities ranging from antioxidant, anti-elastase, anti-collagenase, anti-hyaluronidase, and anti-inflammatory to anti-senescence activity [176]. Some of the studies combined in vitro, ex vivo (using human skin organ culture), and in vivo clinical trials when assessing NP anti-aging activities [177,178]. The advantages of in vitro assays compared to in vivo models include improved reproducibility [176] and a reduction in the number of animals used in research [157].

**Figure 3:**
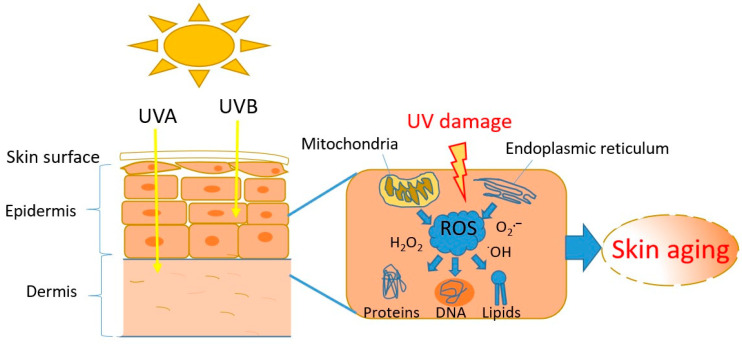
Skin exposure to UV radiation (both, UVA and UVB rays) results in the production and accumulation of ROS such as superoxide (O_2_^•−^), hydroxyl radical (^•^OH), and hydrogen peroxide (H_2_O_2_), leading to oxidative stress and damage to macromolecules (DNA, lipids, proteins), resulting in aging.

The excessive production of ROS in the skin, as the result of UV exposure, leads to ROS accumulation, oxidative stress, and skin aging (i.e., loss of skin elasticity, thinner skin, and more wrinkling) (Figure 3). However, ROS are also produced during regular cellular metabolism, mainly in mitochondria and the endoplasmic reticulum, and are important for redox signalling processes [137,179]. Exposure to damaging UV radiation and other stress factors (e.g., pollution) results in excessive ROS levels that cannot be removed by natural mitigation processes (i.e., antioxidants, enzymes, and pigments), causing elevated intracellular ROS levels and oxidative stress, which leads to cellular damage in macromolecules (i.e., lipids, proteins, and DNA) [39,180]. Cultured keratinocytes, human skin cells making up to 90% of all skin epidermal cells, are used for in vitro testing of anti-aging properties of natural products [153,181]. The effect of targeted NPs and their anti-aging capacities have been assessed using a range of spectrophotometric assays such as antioxidative assays (DPPH and ABTS assays), then a tyrosinase inhibition assay for evaluating anti-pigmentation activity, while collagenase and elastase inhibition assays have been used for confirming the anti-wrinkle activity of NPs [176].

In vitro antioxidative assays such as the DPPH have been used to evaluate the ability of NPs to remove free radicals and protect cells from ROS-induced damage. This assay was used to test MAAs’ ability to shield human keratinocyte cells from UV radiation (300–400 nm) [153]. These HaCaT cells were irradiated with 15 min of UV (275 kJ/m^2^, corresponding to ~90 min of high UV summer sun exposure) and supplemented with various concentrations of MAA (range of: 0, 0.03, 0.15, and 0.3 mM). A strong antioxidative capacity and ability to scavenge free radicals were demonstrated for mycosporine-glycine using a DHHP assay [153]. Similarly, the phosphatidylcholine peroxidation inhibition assay also confirmed strong mycosporine-glycine antioxidative activity [150]. Using a DPPH assay confirmed the ability of polyphenols (i.e., lignans) to scavenge ROS and their antioxidative properties in a concentration-dependent manner [181]. Anti-inflammatory and antioxidant actions of MAP, fucoidans, were confirmed in vitro, but also in vivo using the zebrafish model, indicating strong potential for their addition to cosmetic products [164].

Omega-3 polyunsaturated fatty acids EPA and DHA, using in vitro cultures of HaCaT keratinocyte and CCD922SK fibroblast cell lines, inhibited UVB-induced inflammation by reducing the secretion of IL-8 [182]. In vivo study with healthy human volunteers that used nutritional supplementation of omega-3 over a 10-week period, in the case of EPA, resulted in the reduced production of proinflammatory lipids in UV-exposed skin, while the addition of DHA resulted in lower UV-induced migration of Langerhans cells from the epidermis, which impacts adaptive immune responses [183]. Therefore, more in vivo studies are needed for the improved clinical assessment of the beneficial effects of NPs. However, with ethical limitations and the restricted availability of human volunteers, clinical evidence may take longer than anticipated, and more in vitro models will still be needed to complement pre-clinical trials around the efficiency of NPs in anti-aging processes.

## 5. Proteomics for Natural Product Discovery

Using omics technologies, such as proteomics, has allowed faster discovery and functional characterisation of novel marine natural products and their use as new drug therapies in wound healing and cosmetics [184,185]. The proteome, representing all proteins within cells or organisms, is explored to give a better understanding of proteins’ function and structure, cellular regulatory processes, and protein interactions [186,187]. On the more applied side, the proteomics approaches have been applied to identify specific proteins important in disease detection or development, allocate new drug targets, and follow up on protein expression changes in response to particular conditions/therapies [188]. The more recent interdisciplinary field of chemical proteomics combines proteomics with chemistry to explore and identify drug–target interactions, including the synthesis of probes and target fishing, and better understand the relationship between NPs and drug targets [189].

The application of the proteomic approach in red algae, *Asparagopsis taxiformis*, included the use of different protein extracts from various developmental stages (i.e., sporophyte and gametophyte stages) and provided the most comprehensive overview of the protein profile from the algal development stages [190]. During the process of sample extraction and protein identification, the workflow included the separation of water-insoluble and water-soluble proteins that were separately analysed via high-performance liquid chromatography (HPLC) and mass spectrometry (MS)-based proteomics. In water-soluble and -insoluble fractions and from two protein databases (genome and transcriptome), totals of 741 and 2007 unique non-redundant proteins were identified, respectively. In different extracts, variations in protein profiles were noted, and the set of targeted proteins was identified for further exploration of promising bioactivities. Via this proteomics analysis, 40 secreted proteins were confirmed, including 10 rhodophyte collagen-alpha-like proteins that should be further explored for potential use in anti-aging [190] applications.

In macroalgae, the protein extraction step was identified as a challenging step for the recovery of high-quality MS data due to complex polysaccharides negatively impacting protein purification and downstream proteomic analyses [190,191]. The improved methodology for both high-throughput top-down (i.e., analysis of intact proteins) and bottom-up (i.e., analysis of digested proteins into peptides) proteomic analyses and overcoming these issues has been proposed to include the use of an MS-compatible surfactant, 4-hexylphenylazosulfonate, which was found to facilitate protein extraction and rapid enzymatic digestion, increasing the efficiency of MS-based proteomics analyses [191].

Microalgae have great potential with diverse nutrients that could be applied in the human diet and other industry applications [37]. Peptidomics studies in *Tetradesmus obliquus* identified ~500 bioactive peptides such as angiotensin-converting enzyme (ACE)-inhibitory and antioxidant bioactive peptides with four peptides selected for further analyses [192]. The proteome profile of MAA-producing *Chlamydomonas* species under limited nitrogen conditions contained more proteins with lower N content compared to the control condition, indicating the ability of this microalgae to adapt under stressful starvation conditions [193]. A better understanding of the impact of N status could be used to enhance the biotechnological potential of microalgae for MAA biosynthesis. In the green microalga *Dunaliella parva*, protein expression changes resulted in differential protein levels impacting multiple processes such as photosynthesis, stress response, lipid metabolism, as well as carbohydrate and nitrogen metabolism [194]. Understanding lipid metabolism and its regulation under N-limited conditions is critical in improving ways to enhance the synthesis of valuable NPs such as n-3 PUFA [195]. In the green microalgae *Chlorella vulgaris*, with low copper ions (Cu^2+^), proteome changes impacted fatty acid biosynthesis and the carbon fixation process, with severely reduced protein numbers (from 581 to 369 proteins) and reduced growth [196]. Therefore, proteomics profiling allowed a better understanding of protein changes occurring in metabolic pathways, especially of lipid metabolism and photosynthesis, which are modified during stress, and these are relevant for other biotechnological applications to improve efficient use and control of growth conditions [197]. Many of the isolated bioactive peptides present tremendous potential not only in medicine but also in industry applications and as nutraceuticals in human health [185]. Consequently, a better understanding of lipid metabolism, stress responses, and other metabolic changes using the proteomics approach provides an innovative way to optimise and enhance algal cultivation conditions for the improved industrial production of valuable anti-aging NPs.

## 6. Conclusions

Aging is the inevitable life progression that involves multiple stages, with the gradual deterioration of physiological functions and metabolic processes, and disease development. Slowing aging processes and prolonging the lifespan could be positively influenced by nutritional changes and pharmacological supplements. Anti-aging natural products, including polyphenols, polysaccharides, vitamins, and others, are becoming more popular with the increase in demand due to the rapid rise of the elderly world population. Marine natural products have been shown to have promising anti-aging properties due to their anti-inflammatory, antioxidant, photoprotective, anticancer, and anti-pigmentation activities. Interest in using marine ingredients has steadily increased over the past few decades, with the application of anti-aging natural compounds in various cosmetics products. Marine algae are the most prevalent group found in the marine environment, capable of quicker reproduction and mass growth compared to terrestrial counterparts and consequently more economically valuable. Macro- and microalgae contain beneficial NPs with anti-aging properties, and these biomolecules, by manipulation of external conditions, can be accumulated and produced in mass quantities to facilitate their applications in biotechnology. Advanced technologies of bioinformatics, including proteomics, genomics, transcriptomics, and metagenomics, increased understanding of biosynthetic pathways, genetic polymorphisms, gene regulation, and the best ways to enhance biosynthesis and applications of natural products. Furthermore, omics technologies allowed for enhanced cultivation conditions and improved the understanding and application of these anti-aging products in human health. Finally, marine NPs, as organic and environmentally friendly molecules, are meeting high safety standards and allowing for the innovative use of these compounds with anti-aging properties in various biotechnological applications, promising the sustainable economic growth of this sector in the future.

## Data Availability

Not applicable.
